
JOURNAL INFORMATION
==============================
Journal ID: Wirtschaftsdienst

Wirtschaftsdienst

EISSN: 1613-978X

ARTICLE INFORMATION
==============================
PMCID: PMC8933174
DOI: 10.1007/s10273-022-3129-0
Article ID: 3129
Article version: 1
Subjects: Zeitgespräch

Einkommen und Kreditschulden privater Haushalte

Income and Credit Debt of Private Households

Grabka Markus M. mgrabka@diw.de

Bartz Berenike
grid.8465.f 0000 0001 1931 3152 SOEP, Deutsches Institut für Wirtschaftsforschung, Mohrenstraße 58, 10117 Berlin, Deutschland
Electronic publication date: 2022 Mar 19
Print publication date: 2022
Volume: 102
Issue: 3
First page: 175
Last page: 180
Copyright: © Der/die Autor:in 2022
License: Open Access: Dieser Artikel wird unter der Creative Commons Namensnennung 4.0 International Lizenz veröffentlicht (https://creativecommons.org/licenses/by/4.0/deed.de).
Open Access wird durch die ZBW — Leibniz-Informationszentrum Wirtschaft gefördert.
License URL: https://creativecommons.org/licenses/by/4.0/

Keywords: G51, I39, D31
issue-copyright-statement: © The Author(s) 2022

==============================

Die wirtschaftlichen Auswirkungen der Coronapandemie haben im Jahr 2020 zu einem deutlichen Rückgang des realen BIP in Deutschland geführt. Für Erwerbstätige führte die Krise zu Arbeitsplatzverlusten, Einkommensverlusten – teilweise kompensiert durch Kurzarbeitergeld – und Rückgängen beim Einkommen aus Selbstständigkeit. Während sich die Einkommensseite für die privaten Haushalte damit schwach bis rückläufig in der Krise entwickelte, blieben Verbindlichkeiten davon unberührt und mussten weiter bedient werden. Umso überraschender ist der Befund eines drastischen Rückgangs der Überschuldung in Deutschland im Jahr 2021 (Verband der Vereine Creditreform, 2021).[1] Ziel des vorliegenden Beitrags ist, die Entwicklung der Einkommen und der Kreditbelastung privater Haushalte in Deutschland seit dem Jahr 2000 zu beschreiben. Datengrundlage hierfür ist das Sozio-oekonomische Panel (SOEP) (Goebel et al., 2019), eine seit 1984 jährlich in Deutschland durchgeführte Panelbefragung unter derzeit rund 30.000 erwachsenen Befragungspersonen.

## Einkommen

Seit Juni 2010 ist das Gesetz zur Umsetzung der Verbraucherkreditrichtlinie in Deutschland in Kraft. In diesem Zuge müssen Bankberatende einen Kunden vor Abschluss eines Kreditvertrags sowohl eingehend informieren als auch prüfen, ob ein Kredit für diesen Kunden geeignet ist und seinen finanziellen Möglichkeiten gerecht wird. Insbesondere die Einkommenssituation ist dabei für die Einschätzung der finanziellen Tragfähigkeit eines Haushalts relevant, ob dieser also in der Lage sein wird, die Rückzahlungen auch in der langen Frist zu leisten.

Um die Einkommenssituation privater Haushalte in Deutschland insgesamt zu beschreiben, werden hier retrospektiv für das Vorjahr bedarfsgewichtete Haushaltsnettoeinkommen herangezogen. Als Bedarfsgewicht wird wie in den Berechnungen für den Armuts- und Reichtumsbericht der Bundesregierung die modifizierte OECD-Skala verwendet, nach der einem Haushaltsvorstand ein Gewicht von 1, Kindern bis 14 Jahren ein Gewicht von 0,3 und allen weiteren Haushaltsmitgliedern ein Gewicht von 0,5 zugewiesen wird. Im Jahr 2000 lag das durchschnittliche bedarfsgewichtete Haushaltsnettoeinkommen real bei rund 23.000 Euro und ist bis 2018 – dem derzeit letzten verfügbaren Datenpunkt – auf knapp 26.500 Euro gestiegen. Dies entspricht einem realen Einkommenszuwachs von nahezu 14 %.

Je nach Erwerbsstatus, beruflicher Position oder Alter kann die eigene Einkommensentwicklung stark von der durchschnittlichen abweichen. Um Trends für unterschiedliche Einkommensgruppen zu beschreiben, wird die Bevölkerung nach der Höhe des Einkommens sortiert und in zehn gleich große Gruppen (Dezile) unterteilt. Das unterste (oberste) Dezil gibt somit die Einkommenssituation der ärmsten (reichsten) 10 % der Bevölkerung an.[2] Für eine Betrachtung der prozentualen Veränderung über die Zeit wurden die Einkommen auf das Jahr 2000 (100 %) normiert (vgl. Abbildung 1).

Im Zeitraum 2000 bis 2013 haben sich die Einkommen insgesamt nur schwach entwickelt. Seit 2013 ist ein fahrstuhlartiger Effekt zu beobachten, wonach nahezu alle Einkommensgruppen von steigenden Realeinkommen profitieren. Ab dem Jahr 2015 gilt dies über alle Dezile hinweg. Über die Zeit hinweg haben sich die Einkommensgruppen aber

1 Parallel dazu hat sich die Zahl der Privatinsolvenzen in Deutschland 2021 gegenüber dem Vorjahr auf rund 109.000 Fälle nahezu verdoppelt (Crif, 2022). Dieser Anstieg dürfte mutmaßlich darauf zurückzuführen sein, dass Anträge zuvor zurückgehalten wurden, um von der Reform des Insolvenzverfahrens zu profitieren, nach der die Laufzeit von sechs auf drei Jahre reduziert wird.

2 Zu beachten ist, dass die Personen über die Zeit hinweg aufgrund von Einkommensmobilität ihre Einkommensposition verändern können und nicht immer demselben Dezil zuzuordnen sind.

* Der Beitrag basiert auf einem Vortrag beim Sachverständigenrat für Verbraucherfragen beim BMJV: „Verbraucherverschuldung in den Zeiten von Covid-19“ am 16. Dezember 2021 in Berlin, https://www.svr-verbraucherfragen.de/2021/12/17/verbraucherverschuldung-in-den-zeiten-von-covid-19/ (3. März 2022).

Abbildung 1
**Entwicklung der realen verfügbaren Haushaltseinkommen nach Dezilen**

2000=100

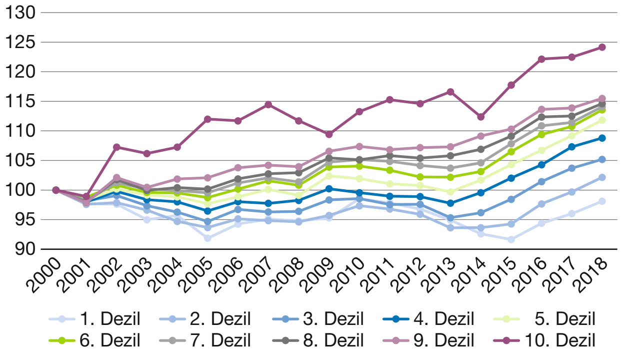

Quelle: SOEPv36. Personen in Privathaushalten. Retrospektiv erfragte Vorjahreseinkommen, bedarfsgewichtet.

Abbildung 2
**Anteil der Privathaushalte mit Kreditschulden**

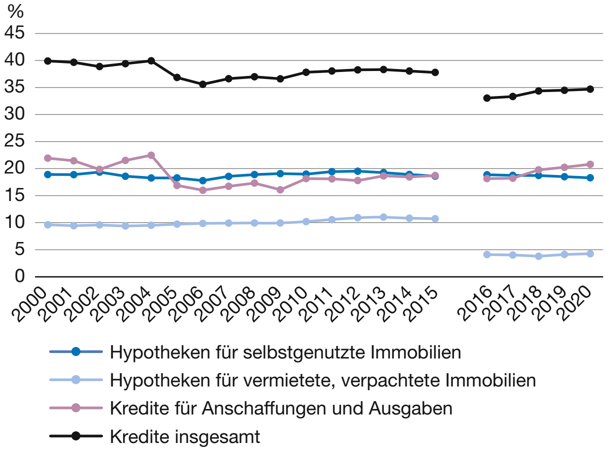

Quelle: SOEPv37 vorläufige Daten. Privathaushalte. Ab 2016 veränderte Abfrage der Hypotheken auf vermietete Immobilien.

ausdifferenziert. Während in der Mitte der Verteilung (5. Dezil) insgesamt ein realer Einkommenszuwachs von 12 % erreicht wurde, stagnieren insgesamt die Einkommen im untersten Dezil, da für diese Einkommensgruppe zwischenzeitlich deutliche Rückgänge zu verzeichnen sind. Anders verhält es sich mit dem obersten Dezil. Die realen Einkommen sind unter Schwankungen bis zum Jahr 2018 um insgesamt etwa 24 % angewachsen.

## Kreditschulden

Mit steigenden (realen) Einkommen eines Haushalts nimmt auch dessen finanzielle Tragfähigkeit zu, sodass sich dieser eher verschulden und Konsum vorziehen kann. Im SOEP werden Kreditbelastungen jedes Jahr auf der Haushaltsebene erfasst. Es werden insgesamt drei Arten von Krediten getrennt erfragt: *Erstens* Hypothekendarlehen auf eine selbstgenutzte Immobilie, *zweitens* Hypothekendarlehen auf vermietete oder verpachtete Immobilien und *drittens* Kredite für Anschaffungen oder sonstige Ausgaben. In der letzten Kategorie wird nicht genauer spezifiziert, um welche Art von Krediten es sich hierbei handelt. Dies können Konsumentenkredite z. B. zum Kauf eines Fahrzeugs oder von Hausrat sein, darunter sind aber auch Kreditkartenschulden, Privatkredite genauso wie Überziehungskredite bei Girokonten oder auch Studienkredite zu verstehen.

Neben kreditbasierten Schulden gibt es weitere Verbindlichkeiten, die im SOEP jedoch nicht erfragt werden. Hierzu gehören beispielsweise Schulden aus nicht gezahlten Rechnungen (Strom, Telekommunikation, Finanzamt, Krankenkassen, Handel), ausstehenden Mietzahlungen sowie Schulden durch Unterhaltsverpflichtungen oder bei Inkassobüros. Dies bedeutet, dass zwar im SOEP kreditbasierte Schulden gut beschrieben sein dürften, das gesamte Ausmaß an Verschuldung aber unterschätzt wird.

Im Jahr 2020 hatte rund ein Drittel aller Privathaushalte Kreditschulden (vgl. Abbildung 2). Während Hypothekenkredite für vermietete oder verpachtete Immobilien nur von weniger als 5 % aufgenommen werden, haben 18 % der Privathaushalte Hypothekenkredite auf selbstgenutzte Immobilien. Kredite für Anschaffungen und Ausgaben werden von etwas mehr als einem Fünftel in Anspruch genommen.

Aufgrund einer veränderten Eingangsfrage bei den Hypotheken auf vermieteten Objekten, wird eine Trendaussage nur für den Zeitraum von 2000 bis 2015 vorgenommen. Über alle Privathaushalte hinweg zeigt sich ein leicht abnehmender Anteil an Krediten. Parallel dazu sind die Zinssätze für Hypothekendarlehen mit beispielsweise einer zehnjährigen Zinsbindung im Zeitraum 2000 bis 2015 von 6,4 % auf nur noch 1,7 % gesunken. Damit ist die Finanzierung von Immobilien – zumindest was die Zinsseite betrifft – deutlich preiswerter geworden (Verband deutscher Pfandbriefbanken, 2019). Auch bei Konsumentenkrediten ist ein Rückgang der Effektivzinssätze zu verzeichnen, der jedoch deutlich schwächer ausfällt.

Um eine erste Aussage über die Gefahr einer möglichen Überschuldung in der Bevölkerung zu geben, wird die Verbreitung von Kreditschulden nach den Dezilen des monatlichen Haushaltsnettoeinkommens analysiert (vgl. Abbildung 3). Es zeigt sich, dass im ersten Dezil 2020 nur 15 % der Haushalte Kreditschulden halten. Mit steigenden Einkommen nimmt die finanzielle Tragfähigkeit

Abbildung 3

**Verbreitung von Kreditschulden nach Dezilen des Haushaltsnettoeinkommens 2020**

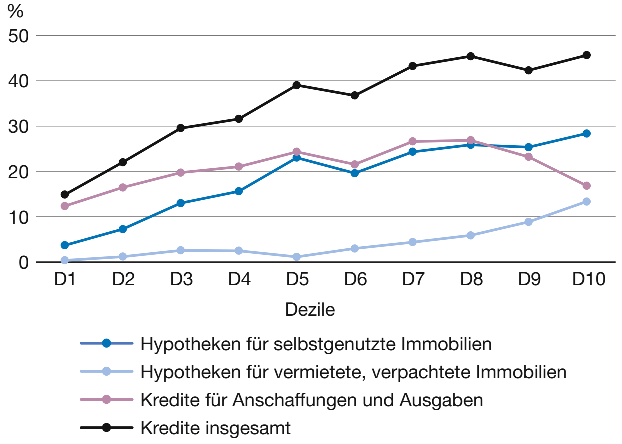

Quelle: SOEPv37 vorläufige Daten. Privathaushalte. Bedarfsgewichtete aktuelle Haushaltsnettoeinkommen.

Abbildung 4

**Durchschnittliche Höhe der monatlichen Kreditrückzahlungen (inklusive Tilgung) in Euro**

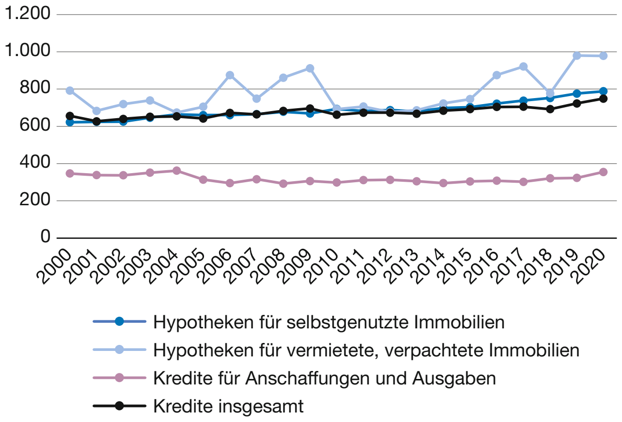

Quelle: SOEPv37 vorläufige Daten. Nur Haushalte mit Kreditschulden.

der Haushalte zu, was sich auch in einer höheren Bereitschaft zur Kreditaufnahme äußert. Im obersten Dezil wird auch der höchste Anteil an Haushalten mit knapp 46 % mit Krediten erreicht. Die Zusammensetzung der Kredite variiert über die Dezile. Während in den ersten vier Dezilen Kredite für Anschaffungen und Ausgaben dominieren, nimmt die Bedeutung von Hypotheken für selbst genutzte Immobilien kontinuierlich über die Einkommensverteilung zu, von knapp 4 % im ersten Dezil bis hin zu 28 % im zehnten Dezil. Im obersten Dezil nehmen die Kredite für Anschaffungen und Ausgaben in ihrer relativen Bedeutung wieder ab, dafür gewinnen Hypotheken auf vermietete Immobilien an Bedeutung und erreichen für diese Einkommensgruppe einen Anteil von 13 %.

Der monatliche Betrag, den ein Haushalt im Durchschnitt aufwendet, um seine Schulden zu bedienen, liegt 2020 bei knapp 750 Euro (vgl. Abbildung 4). Im Falle von Hypotheken umfasst dies sowohl die zu entrichtenden Zinsen als auch die Tilgung. Im Vergleich zum Jahr 2000 hat sich dieser Wert durchschnittlich um etwa 100 Euro im Monat nominal erhöht. Da aber parallel dazu die Effektivzinsen gesunken sind, ist zu folgern, dass die Schuldenhöhe gestiegen sein dürfte. So berichtet das Statistische Bundesamt (2021), dass das Gesamtvolumen aller Kredite und sonstigen Verbindlichkeiten der privaten Haushalte und privaten Organisationen ohne Erwerbszweck im gleichen Zeitraum um rund 30 % auf etwa 1,96 Billionen Euro zugenommen hat.

Die Rückzahlungen für Hypotheken auf vermietete Objekte fallen mit rund 1.000 Euro im Jahr 2020 am höchsten aus, gefolgt von rund 800 Euro für Hypotheken auf selbstgenutzte Immobilien. Für Konsumentenkredite wenden die betreffenden Haushalte dagegen nur etwa 350 Euro im Monat auf. Dieser Betrag hat sich gegenüber dem Jahr 2000 kaum verändert.

Die Höhe der Rückzahlungen kann je Einkommensposition eine unterschiedlich hohe finanzielle Bürde darstellen. Um eine Aussage über die Höhe der Belastung durch Rückzahlungen von Krediten zu treffen, wird der Quotient aus gezahlten Rückzahlungen und laufendem aktuellen Haushaltsnettoeinkommen gebildet. Die durchschnittliche Belastungsquote ist in den vergangenen 20 Jahren von 26 % auf 20 % deutlich gefallen (vgl. Abbildung 5). Dieser Trend liegt für alle Einkommensquintile[3] vor. Die höchste durchschnittliche Belastungsquote liegt erwartungsgemäß für das erste Quintil bei einem Anteil von knapp 24 % vor. In allen anderen Quintilen liegt die Quote bei etwa 20 %. Eine Differenzierung nach dem Wohnstatus zeigt zudem, dass selbstnutzende Eigentümerhaushalte mit einer Belastungsquote von 24 % stärker finanziell aufgrund von Hypotheken belastet sind im Vergleich zu 13 % bei Mieterhaushalten. Über die Zeit hinweg ist die Belastungsquote bei Eigentümer:innen um 8 Prozentpunkte deutlich stärker gesunken als bei Mieter:innen mit rund 3,5 Prozentpunkten.

Betrachtet man die Verteilung der Belastungsquoten über alle Privathaushalte hinweg, so wiederholt sich zunächst der bereits beschriebene Befund, dass knapp zwei Drittel der Haushalte keine Kreditschulden zu tilgen

3 Quintile erhält man, in dem die Bevölkerung nach der Höhe des Einkommens sortiert und in fünf gleich große Bevölkerungsgruppen eingeteilt wird.

Abbildung 5

**Anteil der Rückzahlungen für Kredite am Haushaltsnettoeinkommen**

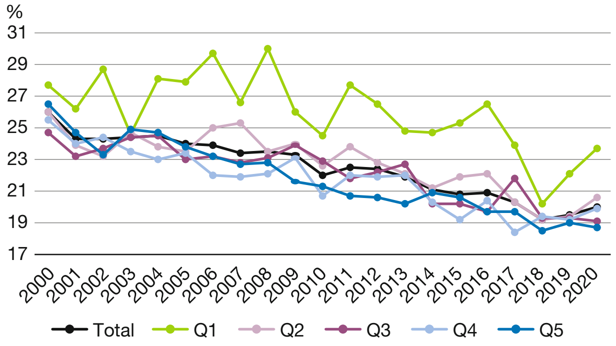

Quelle: SOEPv37 vorläufige Daten. Privathaushalte. Aktuelle Haushaltsnettoeinkommen.

Abbildung 6

**Verteilung der Belastungsquote[1] 2000 bis 2020**

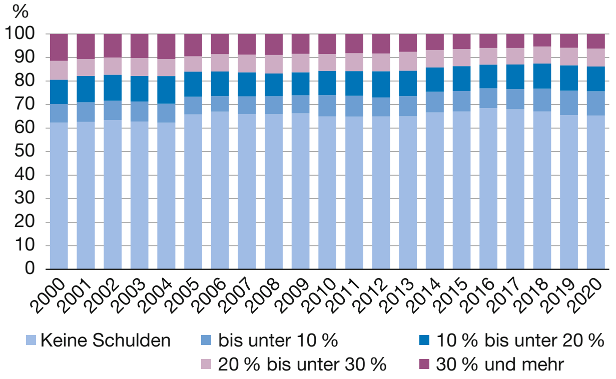

[1] Anteil der Rückzahlungen für Kredite am aktuellen Haushaltsnettoeinkommen.

Quelle: SOEPv37 vorläufige Daten. Privathaushalte.

Abbildung 7

**Anteil der Haushalte mit Risiko der Überschuldung[1] nach Alter des Haushaltsvorstands**

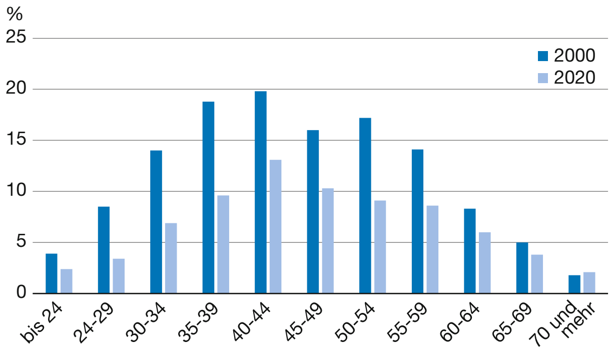

[1] Mehr als 30 % des Einkommens werden für Rückzahlungen aufgewendet.

Quelle: SOEPv37 vorläufige Daten. Privathaushalte.

haben (vgl. Abbildung 6). Eine Belastungsquote von weniger als 10 % des aktuellen Haushaltsnettoeinkommens haben ein Zehntel aller Haushalte zu leisten. Dieser Anteil ist seit 2000 leicht um 2 Prozentpunkte gestiegen. Eine Belastungsquote zwischen 10 % bis 20 % wird wiederum von einem Zehntel aller Haushalte berichtet, wobei dieser Anteil konstant bleibt. Eine weitere Gruppe mit konstantem Bevölkerungsanteil umfasst diejenigen mit einer Belastungsquote zwischen 20 % bis 30 %. Deren Anteil liegt bei etwa 8 % aller Privathaushalte. Diejenigen Haushalte mit der höchsten Belastungsquote von mehr als 30 % des Haushaltsnettoeinkommens ist auch die mit der größten Veränderung. Während im Jahr 2000 noch etwa 12 % aller Haushalte mehr als 30 % ihres Einkommens für Kreditrückzahlungen leisteten, hat sich dieser Anteil bis 2020 halbiert. Das Risiko einer Überschuldung ist demnach in Deutschland in den vergangenen Jahren deutlich gesunken.

Banken empfehlen häufig bei Kreditaufnahme eine 50-30-20 Regel, nach der 50 % des Einkommens die Grundausgaben decken sollte, 30 % für persönliche Bedürfnisse ausgegeben und 20 % zur Schuldentilgung verwendet werden sollten. Liegt die Belastungsquote bei mehr als 30 % des Haushaltsnettoeinkommens kann von einem Risiko der Überschuldung ausgegangen werden. Differenziert man das Risiko der Überschuldung nach dem Alter des Haushaltsvorstands, so liegt ein umgekehrt u-förmiger Verlauf vor (vgl. Abbildung 7).

Bei jungen Erwachsenen ist zunächst das Risiko der Überschuldung gering und steigt bis zur Altersgruppe der 40 bis 44-Jährigen kontinuierlich an. Im Jahr 2020 lag für diese Gruppe der Anteil der vom Risiko der Überschuldung bedrohten bei 13 %. Mit weiter zunehmendem Alter sinkt das Risiko der Überschuldung wieder. Im Vergleich zum Jahr 2000 hat sich am grundsätzlichen Muster wenig verändert, wenngleich das Niveau über alle Altersgruppen hinweg gesunken ist. Auffallend ist aber, dass bei den über 60-Jährigen der Rückgang unterdurchschnittlich ausfiel.

Nach Haushaltstyp differenziert ist wiederum ein rückläufiger Trend des Risikos einer Überschuldung zu beobachten (vgl. Abbildung 8). 2020 weisen Ein-Personen-Haushalte sowie Paare ohne Kinder mit jeweils knapp unter 5 % das niedrigste Risiko einer Überschuldung auf, gefolgt von den Alleinziehenden mit rund 7 % und Paaren mit einem Kind bis 14 Jahren mit 9 % Überschuldungsrisiko.

Ein überdurchschnittliches Risiko der Überschuldung liegt für Paarhaushalte ab zwei Kindern vor. Mit 14 % besteht das höchste Risiko der Überschuldung für Familien mit drei und mehr Kindern bis 14 Jahren. Im Zeitverlauf ist das Risiko einer Überschuldung bei Paaren mit Kindern

Abbildung 8

**Kumulierter Anteil der Haushalte mit Risiko der Überschuldung[1] nach Haushaltstyp**

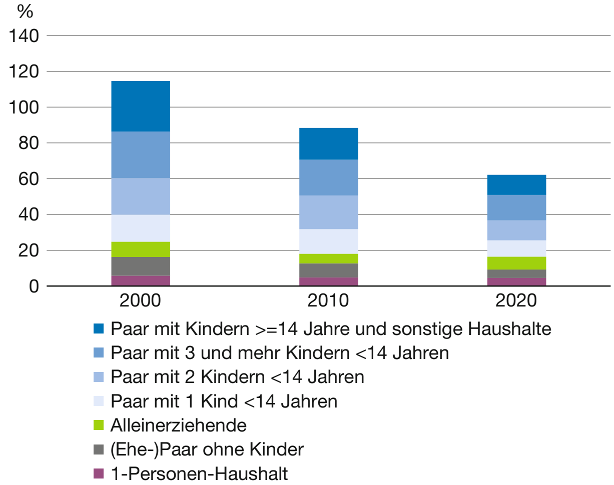

[1] Mehr als 30 % des Einkommens werden für Rückzahlungen aufgewendet.

Quelle: SOEPv37 vorläufige Daten. Privathaushalte.

Abbildung 9

**Empfundene Belastung durch Rückzahlungen für Kredite – Anteil in % mit großer Belastung 2020**

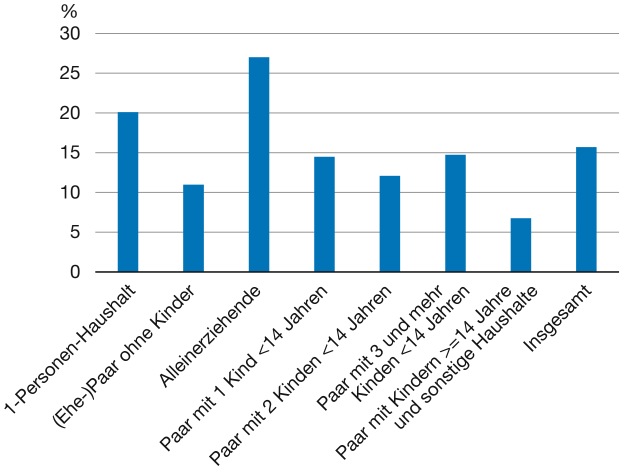

Quelle: SOEPv37 vorläufige Daten. Privathaushalte.

besonders stark gesunken. Dies dürfte auf die insgesamt verbesserte ökonomische Lage dieser Haushalte zurückzuführen sein, da Erwerbslosigkeit zumindest eines erwachsenen Haushaltsmitglieds bei diesen eine geringere Rolle spielt als noch vor rund zehn oder 20 Jahren.

Während das Risiko einer Überschuldung bei Ein-Personen-Haushalten 2020 am geringsten ausfällt, weicht diese von der empfundenen finanziellen Belastung durch Rückzahlungen von Krediten ab. Im SOEP wird die empfundene Belastung mittels einer elfstufigen Skala erhoben, wobei ein Wert von Null bedeutet, dass die Person angibt, die Rückzahlung wäre überhaupt kein Problem, bis hin zu einer zehn mit einer sehr hohen finanziellen Belastung. Im Folgenden wurden die Ausprägungen acht bis zehn zur Identifikation einer hohen empfundenen finanziellen Belastung zusammengefasst (vgl. Abbildung 9). So berichten ein Fünftel aller Ein-Personen-Haushalte mit Krediten, dass die Rückzahlung für sie eine große finanzielle Belastung darstellt. Dies liegt über dem Durchschnitt aller Haushalte mit Krediten mit einem Wert von knapp 16 %. Die höchste Belastung empfinden Alleinerziehende mit einem Wert von 27 %. Im Gegensatz dazu liegt die empfundene Belastung durch Rückzahlungen bei Paarhaushalten unter dem Durchschnitt aller Haushalte mit Krediten.

## Fazit und Ausblick

Im Jahr 2020 hatte rund ein Drittel aller Privathaushalte Kreditschulden. Für Rückzahlungen inklusive eventueller Tilgung wendeten die betroffenen Haushalte im Schnitt knapp 750 Euro pro Monat auf. Dies ist nur rund 100 Euro mehr als vor 20 Jahren. Da im Zeitraum von 2000 bis 2018 die realen verfügbaren Haushaltseinkommen im Durchschnitt um 14 % gewachsen sind, hat parallel dazu die Belastungsquote aus Rückzahlungen im Verhältnis zum Haushaltsnettoeinkommen von im Schnitt 26 % auf 20 % im Jahr 2020 abgenommen.

Eine überdurchschnittlich hohe Belastungsquote von 30 % und mehr hatten im Jahr 2000 noch etwa 12 % aller Privathaushalte. Bis zum Jahr 2020 hat sich dieser Wert nahezu halbiert. Das Risiko einer Überschuldung aufgrund von Kreditschulden ist demnach in Deutschland in den vergangenen Jahren deutlich gesunken. Neben den steigenden Realeinkommen dürfte dabei auch das sinkende Zinsniveau einen wesentlichen Einfluss gehabt haben. Denn bei sinkendem Zins und gegebener Kreditsumme reduziert sich entsprechend der Rückzahlungsbetrag oder alternativ die Rückzahlungsdauer.

Die hier vorgelegten Ergebnisse für das Jahr 2020 sind noch vorläufig. Ungeachtet dessen zeigen sich nach Angaben des SOEP keine grundlegenden Veränderungen durch die Auswirkungen der Coronapandemie auf die Kreditschulden und die Belastungsquoten in Deutschland. Dabei sei aber noch einmal darauf hingewiesen, dass im vorliegenden Beitrag nur Kreditschulden betrachtet wurden. Die Verbindlichkeiten der Privathaushalte gehen aber über die Kreditschulden hinaus und setzen sich z. B. aus unbezahlten Rechnungen (z. B. Mietschulden) zusammen. Auch letztere können zu einer Überschuldung führen. Zudem ist zu beachten, dass Selbstständige neben den hier beschriebenen Schulden weitere Verbindlichkeiten aufgrund ihrer unternehmerischen Tätigkeit haben können. Selbstständige waren von pandemiebedingten Eindämmungsmaßnahmen häufig betroffen, was sich negativ auf deren Geschäft auswirkte (Kritikos et al., 2020). Durch die Aussetzung des Insolvenzrechts ist es bislang zu keiner zunehmenden Zahl von Unternehmensinsolvenzen gekommen. Es ist unklar, was nach Wieder-Inkraftsetzen des Insolvenzrechts passieren wird. Es bleibt aber das Risiko, dass im Falle einer Unternehmensinsolvenz der/die Eigentümer:in je nach Gesellschaftsform auch mit dem Privatbesitz (mit-)haften müssen, sodass auch die Zahl der Privatinsolvenzen in Deutschland wieder zunehmen könnte.

Für Privathaushalte mit Kreditschulden ist eine andere Entwicklung von besonderem Interesse. Vor dem Hintergrund einer anziehenden Inflation, ist nicht auszuschließen, dass die EZB die Nominalzinssätze anheben könnte. Sollte beispielsweise der Nominalzins bei einem neu aufzunehmenden Hypothekendarlehen von 1 % auf 2 % erhöht werden, so hat dies zur Folge, dass der Rückzahlungsbetrag um 25 % steigt. Bei einem Hypothekendarlehen mit einer Höhe von beispielsweise 300.000 Euro und einer Tilgung von 3 %, nähme damit der monatliche Rückzahlungsbetrag von 1.000 Euro auf 1.250 Euro zu.[4] Zwar dürfte die Mehrheit der Hypothekendarlehen eine mehrjährige Zinsbindung haben, sodass ein sich verändernder Nominalzins nur langsam bei bestehenden Kreditnehmenden seine Wirkung entfalten kann, dennoch birgt eine solch nennenswerte Veränderung der Rückzahlungshöhe das Risiko einer zunehmenden Überschuldung in Deutschland.

4 Eine Anhebung des Nominalzinses wirkt sich bei konstanter Tilgung positiv auf die Rückzahlungsdauer aus, die in diesem Beispiel um rund drei Jahre auf 25 Jahre und 8 Monate sinkt.

**Title:** ***Income and Credit Debt of Private Households***

This article describes the development of the needs-weighted net household income of persons in private households in Germany since the year 2000. It also focuses on the presentation of the prevalence and amount of credit debts of private households. The central result is that the share of repayments for loans in Germany has been declining overall since 2000. In particular, the share of households that spend more than 30 % of their household net income on loan repayments has halved over the last 20 years. Despite this overall positive development, there is a risk that the problem of over-indebtedness in Germany could increase again since on the one hand, many self-employed people have suffered high turnover losses due to the containment measures of the coronavirus pandemic, while on the other, the ECB could raise nominal interest rates due to tightened inflation, which would lead to a considerable financial burden for indebted households.

Dr. Markus M. Grabka ist Mitglied im Direktorium des Sozio-oekonomischen Panels (SOEP) und kommissarischer Leiter des Bereichs Wissenstransfer im SOEP am DIW Berlin.

Berenike Bartz ist wissenschaftliche Hilfskraft im Sozio-oekonomischen Panel (SOEP) am DIW Berlin.


Literatur

CRIF (2022), Studie Schuldenbarometer 2021: Fast doppelt so viele private Insolvenzen, https://www.crif.de/pr-events/studien-und-fachartikel/2022/february/17/studie-schuldenbarometer-2021-fast-doppelt-so-viele-private-insolvenzen/ (8. März 2022).
Goebel J The german socio-economic panel (soep) Jahrbücher für Nationalökonomie und Statistik 2019 2 345 360 10.1515/jbnst-2018-0022
Kritikos, A. S., D. Graeber und J. Seebauer (2020), Corona-Pandemie wird zur Krise für Selbständige, DIW aktuell, 47, https://www.diw.de/documents/publikationen/73/diw_01.c.791679.de/diw_aktuell_47.pdf (8. März 2022).
Statistisches Bundesamt (2021), Vermögensbilanzen. Sektorale und gesamtwirtschaftliche Vermögensbilanzen 1999–2020, https://www.de-statis.de/DE/Themen/Wirtschaft/Volkswirtschaftliche-Gesamtrech-nungen-Inlandsprodukt/Publikationen/Downloads-Vermoegens-rechnung/vermoegensbilanzen-xlsx-5816103.xlsx;jsessionid=5679170AC5AD09A774CC9DC9FB0041EE.live741?__blob=publicationFile (8. März 2022).
Verband deutscher Pfandbriefbanken (2019), Hypothekenzinsen historisch niedrig, https://www.pfandbrief.de/site/dam/jcr:48184c24-ba3d-4354-aa7c-22c8d12e6994/Hypothekenzinsen_historisch_niedrig.jpg (8. März 2022).
Verband der Vereine Creditreform (2021), SchuldnerAtlas Deutschland 2021. Überschuldung von Verbrauchern, https://www.boniversum.de/wp-content/uploads/2021/11/CR-S-Atlas-DEU-2021-Bericht.pdf (8. März 2022).
